# Facilitators for establishing a home-based medicine reverse logistic system in low- and middle-income countries – a scoping review

**DOI:** 10.3389/fphar.2025.1568696

**Published:** 2025-07-25

**Authors:** Jose Vincent, Thekkumkara Surendran Anish

**Affiliations:** 1 Department of Community Medicine, Amala Institute of Medical Sciences, Thrissur, Kerala, India; 2 Department of Community Medicine, Nodal Officer, Kerala One Health Centre for Nipah Research and Resilience, Kozhikode, Kerala, India

**Keywords:** medicines, drugs, drug waste, reverse logistic chain, environmental risk

## Abstract

**Background:**

The *end-of-use* and *end-of-life* medicines at households are often disposed of improperly, which has harmful environmental impacts. Health hazards like antimicrobial resistance can occur. A home/household-based medicine reverse logistics system can avoid improper disposal of medicine waste, and it can recover any remaining value from end-of-use medicines.

**Methods:**

We did a scoping review to identify the key facilitators that help the initiation and establishment of a Medicine Reverse Logistics (MRL) system for the collection of unused medicines from households. Google Scholar and PubMed were the databases searched. A qualitative synthesis of included studies was performed. Themes and subthemes were identified. The keywords used were medicines, drugs, reverse logistics, homes, houses, and households.

**Results:**

The awareness level of the various stakeholders, the sharing of responsibilities among stakeholders, incentives to stakeholders, political will and legal framework, and the utilization of logistics managerial capabilities of pharmaceutical companies were identified as key facilitators.

## Introduction

The use of pharmaceutical agents for the prevention and treatment of human or animal disease has been steadily increasing over the years (Kaczala and Blum, 2016; Rzymski, Drewek, and Klimaszyk, 2017). The expansion in human population, the increased prevalence of non-communicable diseases (Boutayeb and Boutayeb, 2005), emerging infectious diseases, improved health access, and longer life expectancy (Chen et al., 2021) can further increase the pharmaceutical waste produced in the coming years (Giakoumakis et al., 2021). In addition to their therapeutic use, pharmaceutical agents like antibiotics are used in livestock as growth promoters (Butaye, Devriese, and Haesebrouck, 2003). Human and animal excretion, improper disposal of unused or expired medication, and waste from pharmaceutical manufacturing plants and hospitals can act as pathways through which pharmaceutical agents can enter the water bodies and soil (Bound and Voulvoulis, 2005; Paut Kustrica et al., 2022). Medicines and pharmaceutical substances are thus emerging pollutants to ecosystems all over the world (Estrada-Almeida et al., 2024). Pharmaceutical agents and their metabolites can negatively impact the ecosystem, cause water contamination, and enter the food chain if they are disposed of improperly (Castensson et al., 2009; Kümmerer, 2009). Many such chemicals are mutagenic and genotoxic (Sharif et al., 2016). Improper use and disposal of medicinal waste have been associated with the incidence of antimicrobial resistance (Tamhankar and Stålsby Lundborg, 2019; Lübbert et al., 2017). Unfortunately, the health threats posed by poorly disposed medicines and far-reaching consequences are not fully understood by the general public and policymakers (Yu et al., 2019). The unused and expired medicines in households are most often disposed of improperly and can act as significant contributors to environmental pollution. (Rogowska and Zimmermann, 2022).

A pharmacological product can become redundant to the consumer if either it has reached the *end-of-use* or has reached its *end-of-life* (Rogowska and Zimmermann, 2022). *End-of-use* in the context of medicines means the patient or consumer no longer needs them, either because the disease has been cured or the patient has been shifted to another regimen. The *end-of-life* means the medicines have reached their expiry. The *end-of-life* and *end-of-use* medicines must be collected back from the end user either to retrieve some value from them or to dispose of them properly (Ribeiro et al., 2021). A supply chain running from the consumer to the manufacturer, known as the medicine reverse logistics (MRL) system, is established to achieve this (de Campos et al., 2021).

The medicine stored in a home environment presents an interesting case. *End-of-use* medicines have the potential to be used by the same members or some other members of the family in another context before their expiry. *End-of-life* medications in households have the risk of being discarded improperly-often with the general waste or in the sewage (Abbas and Farooquie, 2013), and subsequently, impact the environment harmfully. Diverting the *end-of-use* medicines for further use constitutes a social good, as these medicines can be obtained at a subsidized rate (Viegas et al., 2023). This is typically done through regulated channels such as medicine reuse programs or charitable redistribution initiatives, where safety and quality are assured. Even when there are concerns about improper storage and expiration, such initiatives can bring down the carbon footprint and maximize the resource efficiency of the drug manufacturing industry (Cussans et al., 2021). Verification by licensed pharmacists, adherence to strict guidelines on handling and packaging, and limiting redistribution to unopened, properly stored medicines can mitigate many of the risks associated with the redistribution of *end-of-use* medicines. The collection of *end-of-life drugs* from the end consumers ensures that the drugs are properly disposed of (Pereira et al., 2017).

The establishment of a home-based MRL system assumes significance in this backdrop. However, the system for the collection of unused drugs from households is not currently established in most of the Low- and Middle-Income Countries. Recognizing the importance of MRL and identifying systems and processes facilitating its establishment may be pivotal for the health systems of the global south to have such functioning systems in the future. The logistical and managerial aspects of MRL systems that function in high-income countries have been widely studied. However, there exists a theoretical gap in understanding how such systems can be conceptualized, initiated, and sustained in LMICs, where infrastructure, governance, state priorities, and community engagement differ significantly. This review seeks to address that gap by identifying the key facilitators that enable the initiation and establishment of an MRL system for the collection of end-of-use and end-of-life medicines from households in LMICs. Thus, the study contributes to the academic discourse on circular pharmaceutical systems and health systems innovation in resource-limited settings. This can contribute towards a conceptual framework for policy development.

## Methodology

To identify the key facilitators of the establishment of home-based Medicine Reverse Logistics systems, we conducted a systematic search in Google Scholar using the link https://scholar.google.com/scholar?start=20&q=medicine*+OR++drug*++AND++reverse+logistics+AND+home*+OR+house*+OR+household*&hl=en&as_sdt=0,5 and in PubMed using https://pubmed.ncbi.nlm.nih.gov/?term=medicine*+OR+drug*+AND+reverse+logistics+AND+home*+OR+house*+OR+household*. The search terms were created by linking the keywords using Boolean operators. The keywords used were medicines, drugs, reverse logistics, homes, houses, and households. PubMed was chosen for its comprehensive coverage of peer-reviewed biomedical literature, while Google Scholar was included to capture relevant articles not indexed in PubMed. Due to access constraints, we were unable to include other major databases such as Embase, Scopus, or Web of Science.

**FIGURE 1:**
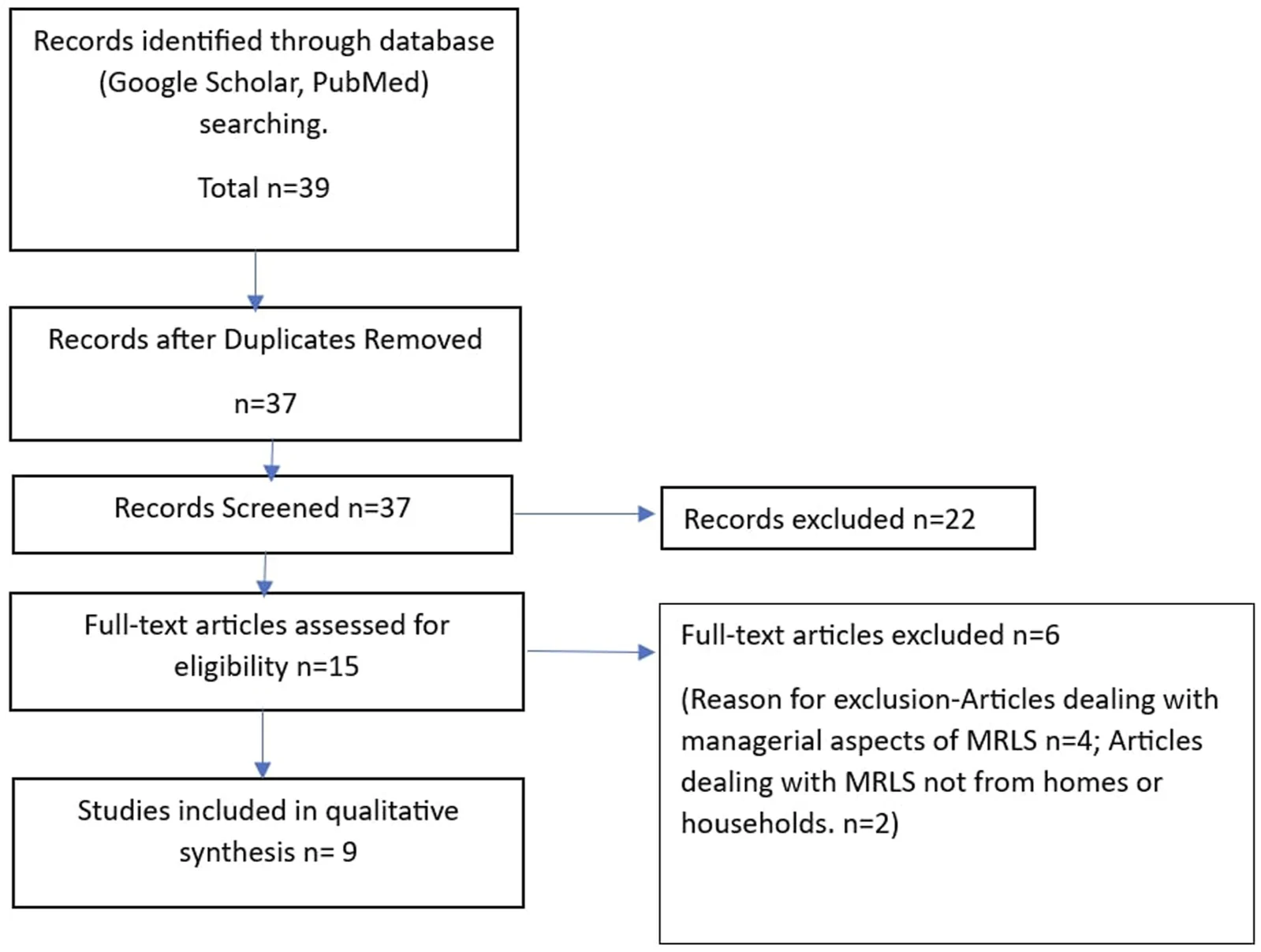
The PRISMA flowchart of the literature search.

Although there were studies dealing with health facility-based MRL systems, we were interested in household-based systems. We did not limit our analysis to original studies because of the scarcity of such studies. We also included systematic reviews, scoping reviews, narrative reviews, qualitative studies, and case studies in our analysis. Expansion of the horizon of our literature search to reviews helped us to extract the findings of other studies not indexed in the databases we used for the current analysis. We could identify 15 articles that dealt with the topic. The authors read through the full articles of these studies. We were on the lookout for key facilitators that would enable the initiation and establishment of an MRL system in a low-middle-income country (LMIC). Yet, we did not restrict our initial search to LMICs. This helped in a comprehensive understanding of existing systems, including those implemented in High-Income Countries (HICs). In High-Income Countries (HICs), such models are more prevalent and at times more mature. We noted that there is a limited number of original studies in the domain of interest. The systems and functions of home-based MRL were addressed in some segments of a few systematic reviews. We identified 9 studies that dealt with some domains of home-based MRL and were included in the final scoping review. The PRISMA flowchart of the literature search is shown in Figure 1.

A qualitative synthesis of the included studies was performed. The list of studies included in the scoping review is given in Table 1. The investigators analysed the full text of the selected articles independently and tagged all the factors facilitating the establishment of a home-based MRLS as ‘codes’. We conducted a qualitative thematic analysis on the codes identified and converted them into themes and subthemes.

**TABLE 1:** List of studies included in the scoping review.

Sl No	Study ID	Title of the study	Type of study	Country/Region
1	Xie and Breen (2014)	Who cares wins? A comparative analysis of household waste medicines and batteries reverse logistics systems: The case of the NHS (United Kingdom) (Xie and Breen, 2014)	Case study	United Kingdom
2	Campos et al. (2017)	Reverse logistics for the end-of-life and end-of-use products in the pharmaceutical industry: a systematic literature review (Campos et al., 2017)	Systematic review	Systematic review
3	Lima et al. (2022)	Medications reverse logistics: A systematic literature review and a method for improving the Brazilian case (Lima et al., 2022)	Systematic review	Systematic review
4	Viegas et al. (2023)	Sustainability Assessment of Medicines Reverse Logistics in Brazil: Outcomes from the National and Local Systems (Viegas et al., 2023)	Case study	Brazil
5	Saravanan and Kumar (2016)	Reverse Logistic Disposal Practices Of Household Pharmaceutical Medicines And Its Impact On Environment In Trichy, Tamilnadu, India (Saravanan and Kumar, 2016)	Cross-sectional study	India
6	Dias et al. (2023)	Reverse logistics of medicines; Case study in the municipality of Belém-Pará (Dias et al., 2023)	Case study	Brazil
7	de Campos et al. (2021)	End-of-use and end-of-life medicines—insights from pharmaceutical care process into waste medicines management (de Campos et al., 2021)	Qualitative study	Brazil
8	Abbas and Farooquie (2013)	Return and Disposal of Unused Medicines: A Customer Perspective of Reverse Logistics (Abbas and Farooquie, 2013)	Cross-sectional Study	India
9	Colasse and Leroy (2024)	Exploring the Barriers and Drivers of Reverse Logistics Implementation: An Embedded Single Case Study In The Belgian Pharmaceutical Supply Chain (Colasse and Leroy, 2024)	Case study	Belgium

## Results

Five themes emerged from our analysis of the factors facilitating the initiation and establishment of a Medicine Reverse Logistics (MRL) system for the collection of *end-of-use* and *end-of-life* medicines from households (Table 2). The government, pharma companies, retailers, pharmacists, and consumers are the key stakeholders (Xie and Breen, 2014) in a reverse logistics system (Campos et al., 2017).

**TABLE 2:** Factors facilitating the initiation and establishment of a home-based medicine reverse logistics (MRL) system.

Sl No	Themes	Subthemes
1	The awareness level of the various stakeholders	1. The awareness of the stakeholders regarding the social benefits of reusing end-of-use drugs (Lima et al., 2022; Xie and Breen, 2014)
		2. The awareness of the stakeholders regarding the environmental good that MRLS offers with regard to the proper disposal of drugs (Lima et al., 2022)
		3. Capacity building of key stakeholders by training (de Campos et al., 2021)
2	The sharing of responsibilities among stakeholders	4. Clearly defined and unambiguous responsibility to all the stakeholders (de Campos et al., 2021; Lima et al., 2022)
		5. Trust and collaboration between stakeholders (de Campos et al., 2021)
		6. Sharing of information among the stakeholders (de Campos et al., 2021)
3	Incentives to stakeholders	7. Availability of a system where the end-of-use drugs could be sold (Campos et al., 2017; Lima et al., 2022)
		8. A support system (financial assistance, inclusion in corporate social responsibility, tax reduction, etc.) for the company to run a home-based MRLS (Xie and Breen, 2014; Viegas et al., 2023)
		9. Compensate the additional workload of pharmacists in establishing a home-based MRLS (Viegas et al., 2023)
4	Legal framework and governance	10. Availability of a federal law or framework for MRLS (Viegas, 2023)
		11. Decentralized planning to ensure the efficiency of home-based MRLS (Viegas, 2023)
		12. Availability of a legal framework making the end-user responsible for the disposal (Xie and Breen, 2014)
		13. Looping the producers in the legal framework of the safe disposal of drugs (Xie and Breen, 2014)
5	Utilization of logistics and managerial capabilities of pharmaceutical companies	14. Using the drug delivery systems for the backflow of drugs (Lima et al., 2022; Xie and Breen, 2014)
		15. Use of technology (e.g., RFID tagging to track drug packages) (Campos et al., 2017)

Awareness of these stakeholders about the importance of an MRLS and the social and environmental harm associated with improper disposal of them emerged as the first theme in our analysis. Often, the stakeholders are unaware of the environmental harm the medicines cause (Lima et al., 2022), and such a lack of awareness can hamper efforts to establish an MRL system. The identification of environmental contamination of medicines as a public health problem can shape public opinion and exert pressure on governments to frame policies reducing the wastage of medicines and subsequent establishment of MRLS (Xie and Breen, 2014). Pharmacists’ awareness about the impact of improper disposal on the environment was associated with whether pharmacists assented to have their pharmacies as collection points for take-back programs (Campos et al., 2017). Well-trained managerial resources having an overall understanding of the system are also essential (de Campos et al., 2021).

‘Sharing the responsibilities among stakeholders’ and the ‘incentives to them’ in participating in such a system were the second and third themes, respectively. The sharing of responsibilities is a cornerstone normative principle of MRL systems (Viegas, 2023, Bond, Pontes, Korzenowski, Bordin, R. dos S. Rosa, et al., 2023). The duties of the stakeholders should be spelled out unambiguously (de Campos et al., 2021; Lima et al., 2022). There should be trust and collaboration between stakeholders, and the sharing of information among the stakeholders is important (de Campos et al., 2021).

There is not much market value to be extracted from expired drugs. Unused medicines recovered before expiry can be sold by the pharmaceutical company at a secondary market at a subsidized rate (Campos et al., 2017; Lima et al., 2022). The operational and managerial costs of running a reverse logistics system can be costly for the company. The Brazilian experience shows that the pharmacists’ workload increased following the establishment of MRLS (Viegas et al., 2023). Consumers will not get any financial gain by returning the drugs. Whether the various stakeholders need to be incentivized and to what extent and by whom are grey areas as far as MRLS is concerned.

‘Legal framework and governance’ emerged as the fourth theme. Strong political will and legal frameworks are crucial in the initiation of the process of establishing an MRLS (Viegas, 2023). Brazil is a country with a well-established Medicine reverse logistics system at the national and municipal levels. A national decree in 2020 led to the establishment of a nationwide federal MRLS in Brazil (Viegas, 2023), which mainly deals with the collection and proper disposal of end-of-use medicines. In contrast, the municipal system lacks such a federal decree, and it lacks many of the procedural capabilities of the national level system (Viegas, 2023). The United Kingdom has a system that imposes fines on the stakeholders if the target level of return of medicines is not achieved (Xie and Breen, 2014). The state might also have to make additional investments in developing infrastructure for medicine storage, human resource development, capacity building, and transportation.

‘Utilization of logistics managerial capabilities of pharmaceutical companies’ is the fifth theme. The pharmaceutical companies have well well-established system for drug delivery to the consumers (Lima et al., 2022; Xie and Breen, 2014), which is constantly upgraded and optimized (Xie and Breen, 2014). This technological and managerial sophistication can be used to make the MRLS system more efficient (de Campos et al., 2021). The use of Radio Frequency Identification (RFID) tagging to track drug packages is an example of using new technologies for the smooth running of MRL systems (Campos et al., 2017). The volume of medicines unused can also be calculated using technology-aided monitoring systems (Campos et al., 2017). This can help in making informed choices regarding prescription practices. The availability of infrastructure facilities is identified as an important facilitator in the establishment and sustenance of MRL systems (de Campos et al., 2021).

## Discussion

The study intended to identify the key factors facilitating the initiation and establishment of a home-based Medicine Reverse Logistics (MRL) System. The awareness of the various stakeholders regarding the harmful effects of improperly disposed of drugs was identified as an important factor leading to the establishment of the MRL system (Aquino et al., 2018). This provides the basis upon which the involvement of all stakeholders in the MRL system can be ensured (Xie and Breen, 2014). It can shape their behavior and increase receptiveness to reverse logistics mechanisms (Lima et al., 2022). The consumers might abstain from harmful drug disposal practices; the pharmacists might be more willing to offer drug collection services at their pharmacies; pharma companies may be willing to invest in safe drug recovery and safe disposal initiatives if the awareness about the problem is established in the community (Lima et al., 2022). Thus, awareness of various stakeholders is pivotal in the establishment and running of a home-based MRL system (de Campos et al., 2021).

Closely linked to awareness is the sense of shared ownership and accountability for proper pharmaceutical waste disposal. MRL systems work on the principle of shared responsibilities (de Campos et al., 2021). The patients will have the responsibility to return medicines; pharmacists have the responsibility to collect medicines and educate their clients. As manufacturers, the pharmaceutical companies have the legal and ethical responsibility to mitigate the environmental damages due to drugs (Malmqvist et al., 2023). The pharma companies can bring to the table their expertise in running forward logistics systems (Liu et al., 2020). Thus, the emergence of formal and non-formal division of labor between the various stakeholders is a key component that salvages the community from the adverse environmental and health impacts of improper household drug disposal (Phougat et al., 2025). Such cooperative structures guarantee that the burden of managing pharmaceutical waste from households is not placed on a single stakeholder but is more evenly distributed across the system. The MRL systems offer a great opportunity for public-private partnerships. The logistical and managerial capabilities of the pharmaceutical industry in running reverse logistics systems can be used to optimize home-based MRL systems (Campos et al., 2017). MRL systems could be featured in the corporate social responsibility schemes of pharma companies (de Campos et al., 2021). Thus, sharing of responsibilities and hand-holding emerges as an important factor facilitating the initiation and establishment of a home-based Medicine Reverse Logistics (MRL) System.

The strong legal, policy, and monitoring statutes that the governments and state authorities institute have a very important role in the establishment and sustenance of MRL systems (Viegas, 2023; Cussans et al., 2021). The social good of MRL systems, the tangible health, and environmental impacts of improperly disposed of drugs necessitate government intervention. The *end-of-use* drugs can offer medical treatment to other individuals at a subsidized rate (Xie and Breen, 2014). Governments in resource-poor settings should view MRL systems as a means of promoting equity in healthcare access. Highlighting this aspect can also promote participation and the shouldering of responsibilities by other stakeholders (Viegas et al., 2023).

A broader public discourse around public health and environmental aspects of the improper disposal of *end-of-use* and *end-of-life* drugs should warrant government interventions (Castensson et al., 2009; Kümmerer, 2009). Such a public discourse also influences the public perception regarding the hazards of pharmaceutical waste, and they may perceive the establishment of the MRL system as a felt need (Althagafi et al., 2022. The public health and environmental advocacy groups can capitalize on this to demand the establishment of strong MRL systems (Althagafi et al., 2022; Alnahas et al., 2020; Lago et al., 2022).

Brazil is touted as an example where strong legal statutes help in the establishment of a nationwide MRL system (Rebehy et al., 2019). The nationwide Brazilian MRL system ensures drugs are disposed of without many environmental hazards. Brazil also has a municipal-level MRL system, which focuses on using end-of-use drugs (Viegas, 2023).

The scale of operation of the MRL system is also important. Brazil began the process at the city or municipality level (Viegas et al., 2023). Subnational or city-wise pilot programs can be started in the initial phase. These can be scaled up later to the national level. India, which does not have an MRL system at any level, can also explore the possibility of piloting one in any of the many national disease control programs it runs (Kumar and Saravanan, 2016).

The governments should supplement strong legal statutes with an incentive system that ensures the sustenance of the home-based MRL system (Desai et al., 2022). The recovery of medicines offers not much financial gain to the pharmaceutical companies (da Silva et al., 2022). There is a definite cost associated with the running of reverse logistics systems. The consumer might have to incur costs, which can reduce the long-term gains of pharmaceutical companies and potentially disincentivize their involvement. (Hua et al., 2016). The government can incentivize the pharma companies’ involvement in MRL systems by tax incentives. Financial gains accrued through the return of drugs can incentivize consumers to return drugs (de Campos et al., 2021). Pharmacists might have to perform additional duties of patient education and inventory management. Framing an incentive system in conjunction with the MRL system will be equally important.

Our review could identify the key factors facilitating the initiation and establishment of a home-based Medicine Reverse Logistics (MRL) System. The government or state authority thus emerges as a key stakeholder in the initiation and establishment of an MRL system. The legal and policy frameworks underpinning the MRL system also fall under the ambit of the governments or state authorities. The awareness level and execution of shared responsibilities of other stakeholders are also important. The fashioning of an incentive system can be very helpful in running the system. Then, there is always a large room for cooperation and handholding between state authorities and pharma companies when it comes to the logistical and managerial aspects of running the MRL system (Lima et al., 2022; Xie and Breen, 2014). These factors are consistent with those identified in other settings like the battery industry (Xie and Breen, 2014) and facility-based MRL systems (de Campos et al., 2021).

### Limitations

The infrastructure, government priorities, and the rules governing the pharma industry can vary from country to country. This review only offers a very broad framework that can facilitate the initiation and establishment of an MRL system. This review only offers a very broad framework that can facilitate the initiation and establishment of an MRL system. Also, we could access only the studies published in PubMed and Google Scholar. Hence, the conclusions made may be vulnerable to selection bias.

## Conclusion

Improper disposal of unused medicines can have devastating health and environmental consequences. It should be viewed as a public health and environmental problem. *End-of-use* drugs and *end-of-life* drugs at home can be disposed of improperly. It can significantly contribute to environmental contamination by pharmaceutical agents. The establishment of a home-based MRL system can be a potential solution. The scoping review identified the awareness level of the various stakeholders, political will and legal framework, the sharing of responsibilities among stakeholders, the utilization of logistics managerial capabilities of pharmaceutical companies, and Incentives to stakeholders as key facilitators. The raised awareness among all the stakeholders can initiate the process of establishing a home-based MRL system. The environmental and health impact of improperly disposed pharmaceutical agents should become part of the public conscience and discourse. This helps all stakeholders to take stances that are conducive to the initiation and sustenance of a home-based MRL system. Incentivizing the involvement of stakeholders is an important factor. Governments have an important role in creating an incentivized environment and laying down normative frameworks.

## Data Availability

The original contributions presented in the study are included in the article/supplementary material, further inquiries can be directed to the corresponding author.
